# Altered structural network architecture is predictive of the presence of psychotic symptoms in patients with 22q11.2 deletion syndrome

**DOI:** 10.1016/j.nicl.2017.07.023

**Published:** 2017-07-26

**Authors:** Maria C. Padula, Elisa Scariati, Marie Schaer, Corrado Sandini, Marie Christine Ottet, Maude Schneider, Dimitri Van De Ville, Stephan Eliez

**Affiliations:** a Developmental Imaging and Psychopathology Laboratory, Department of Psychiatry, University of Geneva School of medicine, Geneva, Switzerland; b Medical Image Processing Lab, Institute of Bioengineering, Ecole Polytechnique Fédérale de Lausanne, Lausanne, Switzerland; c Department of Radiology and Medical Informatics, University of Geneva, Geneva, Switzerland; d Department of Genetic Medicine and Development, University of Geneva School of medicine, Geneva, Switzerland

**Keywords:** Psychosis, Diffusion tensor imaging, Graph theory, Limbic system, Anterior cingulate cortex, Multivariate

## Abstract

22q11.2 deletion syndrome (22q11DS) represents a homogeneous model of schizophrenia particularly suitable for the search of neural biomarkers of psychosis. Impairments in structural connectivity related to the presence of psychotic symptoms have been reported in patients with 22q11DS. However, the relationships between connectivity changes in patients with different symptomatic profiles are still largely unknown and warrant further investigations.

In this study, we used structural connectivity to discriminate patients with 22q11DS with (*N* = 31) and without (*N* = 31) attenuated positive psychotic symptoms. Different structural connectivity measures were used, including the number of streamlines connecting pairs of brain regions, graph theoretical measures, and diffusion measures. We used univariate group comparisons as well as predictive multivariate approaches.

The univariate comparison of connectivity measures between patients with or without attenuated positive psychotic symptoms did not give significant results. However, the multivariate prediction revealed that altered structural network architecture discriminates patient subtypes (accuracy = 67.7%). Among the regions contributing to the classification we found the anterior cingulate cortex, which is known to be associated to the presence of psychotic symptoms in patients with 22q11DS. Furthermore, a significant discrimination (accuracy = 64%) was obtained with fractional anisotropy and radial diffusivity in the left inferior longitudinal fasciculus and the right cingulate gyrus.

Our results point to alterations in structural network architecture and white matter microstructure in patients with 22q11DS with attenuated positive symptoms, mainly involving connections of the limbic system. These alterations may therefore represent a potential biomarker for an increased risk of psychosis that should be further tested in longitudinal studies.

## Highlights

• Altered network architecture discriminates psychotic patients with 22q11DS;
• Altered diffusivity measures are evident in psychotic patients with 22q11DS;
• White matter alterations associated to psychosis are located in limbic regions.

## 1 Introduction

Neuroimaging represents a non-invasive technique with the potential of revealing brain alterations related to psychiatric disorders and to provide neural biomarkers that may serve as diagnostic/prognostic tools. Despite the vast number of studies comparing measures of brain integrity in patients with and without psychosis, brain alterations have not shown enough sensitivity and specificity to be used as valuable biomarkers, thus warranting further investigations.

Patients with 22q11.2 deletion syndrome (22q11DS) are genetically exposed to a high risk of developing schizophrenia (Murphy et al., 1999, Schneider et al., 2014a). Therefore, 22q11DS represents a valuable model for the study of neural biomarkers of psychosis. Furthermore, as all the patients share the same genetic deletion, the syndrome represents a homogeneous model of schizophrenia (Bassett and Chow, 1999). A number of brain alterations have been identified in patients with 22q11DS in association to psychotic symptoms. For instance, we found reduced thickness in frontal and fusiform/lingual cortices in patients with 22q11DS and schizophrenia compared to patients without psychosis (Schaer et al., 2009). Furthermore, a recent study showed that impaired trajectories of cortical development in frontal and parietal regions were associated to increased severity of psychotic symptoms (Radoeva et al., 2016). Gothelf et al. further showed that white and grey matter volumes alterations in regions including the prefrontal cortex predicted the development of psychotic symptoms in patients with 22q11DS (Gothelf et al., 2011).

As recently reviewed by Scariati et al., functional and structural connectivity alterations are also associated to psychotic symptoms in patients with 22q11DS. The majority of the reviewed studies used diffusion measures computed either at each voxel, or along white matter tracts. Commonly used diffusion measures were fractional anisotropy (FA), axial (AD) and radial diffusivity (RD) (Armitage and Bastin, 2000, Budde et al., 2008, Budde et al., 2009, Hagmann et al., 2006, Song et al., 2003). Reduced FA in the uncinate fasciculus, inferior longitudinal and inferior fronto-occipital fasciculi (ILF, IFOF), corpus callosum and cingulum bundle was associated to increased schizotypal traits or positive, negative and general psychotic symptoms (da Silva Alves et al., 2011, Sundram et al., 2010). Other studies found a positive correlation between FA and symptoms severity in the anterior limb of the internal capsule (Perlstein et al., 2014) and in the cingulum bundle (Kates et al., 2015). Reduced AD in the IFOF (Jalbrzikowski et al., 2014) and reduced RD in anterior limb of the internal capsule, uncinate fasciculus (Perlstein et al., 2014) and cingulum bundle (Kates et al., 2015) were also associated to higher positive symptoms severity.

Alternatively, a number of studies employed tractography techniques. Tractography allows the reconstruction of connectivity matrices containing the number of streamlines connecting pairs of brain regions (Bammer et al., 2003, Daducci et al., 2012, Hagmann et al., 2003). These matrices can be used to calculate graph theoretical measures, which give information about the structural network architecture (Hagmann et al., 2008, Sporns et al., 2005). When investigating the integrity of the structural network using graph theory, we observed impairments in patients with 22q11DS compared to controls, in association to increased severity of positive (hallucinations, (Ottet et al., 2013b)) and negative symptoms (Váša et al., 2016).

The findings reported in patients with 22q11DS are similar to those observed in schizophrenia, ultra-high risk (UHR) of psychosis and schizotypal disorder. In schizophrenia, initial studies pointed to a predominant impairment of fronto-temporal connections (Pettersson-Yeo et al., 2011). Furthermore, white matter alterations in the limbic system have been shown (for instance (Bracht et al., 2014, Fitzsimmons et al., 2014, Oestreich et al., 2016). A more recent investigation further suggested the presence of widespread patterns of disconnectivity in schizophrenia, which would involve all of the four lobes (Klauser et al., 2016). When investigating network integrity, altered nodal degree and network efficiency was reported ((Zalesky et al., 2011)) as well as altered rich club connectivity, suggesting alterations in brain hubs (van den Heuvel et al., 2013).

As reviewed in (Vijayakumar et al., 2016) findings in patients at risk of psychosis are instead more controversial, with some studies showing no differences between at risk individuals and healthy controls and others pointing to impairments in fronto-temporal and/or fronto-limbic connections, particularly involving association fibers. Similarly, few studies have been conducted in patients with schizotypal disorder, and showed impairments in the uncinate and fronto-occipital fasciculus and in the cingulate gyrus (DeRosse et al., 2015, Rosell et al., 2014). Fewer studies have investigated differences in white matter integrity in other populations of patients expressing psychotic symptoms, such as psychotic depression (O'Connor and Agius, 2015) and Parkinson's disease (Zhong et al., 2013), but they also seem to point to alterations in frontal and limbic brain regions in association to higher symptoms severity.

In this study, we aimed at investigating white matter alterations in patients with 22q11DS expressing high and low positive symptoms scores. Indeed, studies conducted to date in the 22q11DS population compared patients with 22q11DS to healthy controls, or conducted post-hoc correlation analyses between connectivity measures and symptoms scores. In order to disentangle which brain connectivity alterations are associated to psychosis, it is essential to compare patients with 22q11DS with different levels of symptoms severity. To date, only one study investigated structural connectivity differences in subgroups of patients with 22q11DS (Kikinis et al., 2016). The authors compared adolescent patients with 22q11DS with high and low psychotic symptoms scores and showed altered white matter diffusivity in the most symptomatic patients (Kikinis et al., 2016). However, this investigation was limited by the low number of patients included (*N* = 9 patients with high symptoms severity).

Here, we propose a comprehensive evaluation of the white matter alterations associated to higher psychotic symptoms in 22q11DS including: 1) mean measures of diffusion (FA, AD, RD) along tracts of interest, 2) number of streamlines, obtained with tractography, connecting pairs of brain regions and 3) graph theoretical analysis of the brain network. The above-mentioned measures were compared between the groups of patients with 22q11DS with high and low positive symptoms using common mass-univariate statistical group comparisons (*t*-test) as well as using predictive multivariate approaches (i.e., pattern recognition). The univariate comparison was conducted in order to identify regional differences between the two groups in: 1) diffusion measures computed in specific white matter tracts, 2) number of streamlines connecting pairs of brain regions, 3) graph theory measures. The multivariate investigation was alternatively conducted on the same three measures separately in order to provide a first evidence that different subgroups of patients with 22q11DS can be discriminated based on their pattern of brain connectivity and orient future investigations. More in details, we aimed at identifying patterns of 1) altered diffusion measures in defined white matter tracts, 2) impaired number of white matter streamlines, 3) alterations in graph theory measures, that optimally discriminate the presence of psychosis. Indeed, multivariate analysis techniques are more sensitive to inter-regional relationships (Davatzikos, 2004) as they do not assume independency between the connections. Furthermore, univariate tests only allow inference at the group level, while subject-level predictions are needed for potential biomarkers to prove clinically useful.

We hypothesize that multivariate methodologies will be more sensitive to capture subtle patterns of brain alterations associated to prodromal psychotic symptoms. Furthermore, according to the previous findings, we expect to find patterns of disconnectivity in frontal, temporal and limbic pathways in patients with more severe psychotic symptoms.

## 2 Material and methods

### 2.1 Participants

Patients with 22q11DS were collected in the context of an ongoing longitudinal study (Maeder et al., 2016, Schaer et al., 2009) through announcements in regional parents' associations and through word of mouth. After visual inspection of motion artifacts, a cross-sectional group of 100 subjects with good quality DTI images, aged from 10 to 35 years, was initially selected. Patients were classified according to their score on the positive subscale of the Structured Interview of Prodromal Symptoms (SIPS) (Miller et al., 2002). Patients with a score ≥ 3 on at least one item of the positive subscale (P1-P5) were classified as having at least attenuated positive symptoms of psychosis. Time and frequency criteria were not taken into account in this classification. The same criterion was adopted in our previous study on resting-state fMRI connectivity (Scariati et al., 2014). Thirty-one patients manifested at least attenuated positive symptoms of psychosis at the time of testing and were defined *psy+*. As reported in Supplementary Fig. 1, the majority of patients presented hallucinations (80%); a similar percentage presented delusion (50%) and persecutory ideas (44%), while only a minority of patients presented grandiose ideas and disorganized communication (3% and 16% respectively). These results are in line with previous findings in the same cohort of patients (Schneider et al., 2014b).

As control group, 31 patients individually matched for age and gender were selected and were defined *psy-*. Demographic information for the two groups of patients with 22q11DS are reported in Table 1 and in Supplementary Fig. 2.

**Table 1:** Demographic information.

	*psy+*	*psy-*	*p* value
N. subjects (females)	31 (15)	31 (15)	
Mean age (range)	17.86 ± 5.9 y.o.	17.34 ± 5.5 y.o.	0.7
	(10.28–34.34)	(10.13–31.48)	
Right handed (%)^a^	80.6%	77.4%	0.75
Mean IQ	69.2 ± 11.8	72.8 ± 12.9	0.26
N. subjects meeting criteria for psychiatric diagnosis	24 (77.4%)	19 (61.3%)	0.26
Anxiety disorder	4 (13%)	8 (25.8%)	
Attention deficit hyperactivity disorder	1 (3.2%)	4 (13%)	
Mood disorder	1 (3.2%)	0	
Schizophrenia	2 (6.5%)	0	
Psychotic disorder	2 (6.5%)	0	
More than one psychiatric disorder	14 (45%)	7 (22.6%)	
N. subjects medicated	11 (35.5%)	2 (6.5%)	0.005
Methylphenidate	2 (6.45%)	1 (3.2%)	
Antidepressants	1 (3.2%)	1 (3.2%)	
Antipsychotics	4 (13%)		
Anticonvulsants	2 (6.45%)		
Anxiolytics	0		
More than one class of medication	2 (6.45%)		
SIPS positive scores	9.26 ± 5.7	2.6 ± 2.1	< 0.001
P1	2.8 ± 1.8	0.48 ± 0.6	< 0.001
P2	2.55 ± 1.6	1 ± 0.7	< 0.001
P3	0.32 ± 0.79	0.03 ± 0.2	< 0.001
P4	3.3 ± 1.5	0.68 ± 0.9	< 0.001
P5	1.2 ± 1.5	0.35 ± 0.8	0.02

P1 = unusual thoughts/delusional ideas, P2 = suspiciousness/persecutory ideas, P3 = grandiosity, P4 = perceptual abnormalities/hallucinations, P5 = disorganized communication.

a Handedness was measured using the Edinburgh laterality quotient.

IQ was measured using the Wechsler Intelligence Scale for Children (version III or IV) or the Wechsler Adult Intelligence Scale (version III or IV) (Wechsler, 1991, Wechsler, 1997, Wechsler, 2004, Wechsler, 2008). The average IQ did not significantly differ between the groups of patients with and without psychotic symptoms (*p* = 0.26).

The presence of a psychiatric diagnosis was assessed using the Diagnostic Interview for Children and Adolescents Revised (DICA-R (Reich, 2000)), the psychosis supplement from the Kiddie-Schedule for Affective Disorders and Schizophrenia Present and Lifetime version (K-SADS-PL (Kaufman et al., 1997)) and the Structured Clinical Interview for DSM-IV Axis I Disorders (SCID-I (First et al., 1996)). Twenty-four (77.4%) *psy+* patients and 19 (61.3%) *psy-* patients met the criteria for a psychiatric diagnosis (Table 1). Eleven patients within the *psy+* group (35.5%) and two patients in the *psy-* group (6.5%) were under medication at the time of testing (Table 1).

Written informed consent was received from the patients and their parents and our protocols were approved by the cantonal ethic commission of research.

### 2.2 MRI acquisitions

T1-weighted and Diffusion Tensor (DTI) images were acquired using a Siemens Trio (*N* = 43, 22 *psy+,* 26 *psy-)* or a Siemens Prisma (*N* = 11, 9 *psy+,* 5 *psy-*) 3 Tesla MRI scanner at the Center for Biomedical Imaging (CIBM) in Geneva. The T1-weighted sequence was acquired with a 3D volumetric pulse, TR = 2500 ms, TE = 3 ms, flip angle = 8°, acquisition matrix = 256 × 256, field of view = 23.5 cm, slice thickness = 3.2 mm, 192 slices. DTI images were acquired with the following parameters: number of directions = 30, b = 1000 s/mm^2^, TR = 8800 ms, TE = 84 ms, flip angle = 90°, acquisition matrix = 128 × 128, field of view = 25.6 cm, GRAPPA acceleration = 2, 64 axial slices, slice thickness = 2 mm. The head coil differed between the two scanners (12 channels for the Siemens Trio and 20 channels for the Siemens Prisma).

### 2.3 Data analysis

**Fig. 1:**
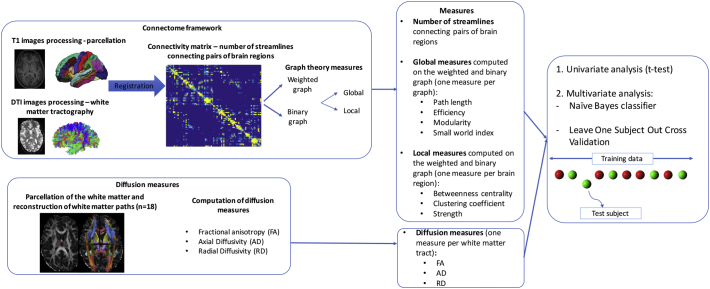
Overview of the data processing and analysis.

Fig. 1 summarizes the different connectivity measures included in the analyses. Three measures of white matter integrity were used:

○ Connectivity matrices containing number of streamlines connecting pairs of brain regions;
○ Graph theory measures, computed on the weighted and binary connectivity matrices;
○ Diffusion measures (FA, AD, RD).

The methods for the computation of each of these measures and the statistical analyses carried out are detailed in the following sections.

#### 2.3.1 Construction of the connectivity matrices

The pipeline for the construction of the connectivity matrices is represented in Fig. 1. First, T1-weighted images were processed using *FreeSurfer* (http://freesurfer.net). The images were preprocessed following previously described steps (resampling into cubic voxels, intensity normalization, skull stripping, tissue segmentation) to extract the white and sub-cortical grey matter volumes and the cortical surfaces (Dale et al., 1999). A parcellation of the entire grey matter was then obtained (Desikan et al., 2006), defining 83 cortical and sub-cortical regions.

DTI images were preprocessed using the FSL Diffusion Toolbox (http://fsl.fmrib.ox.ac.uk/fsl/fslwiki/). After brain extraction and eddy currents distortion correction the effect of geometric distortions was corrected by registering the DTI images to the T1-weighted images using the software ANTs (Advanced Normalization Tools (Avants et al., 2011)). Motion parameters were extracted using the TRActs Constrained by UnderLying Anatomy toolbox (TRACULA (Yendiki et al., 2014), https://surfer.nmr.mgh.harvard.edu/fswiki/Tracula and are reported in the Table 2. Average translation and rotation did not significantly differ between *psy+* and *psy-* patients (*p* > 0.2).

**Table 2:** DTI movement parameters.

	*psy*+	*psy*-	*p* value
Translation (mm)	0.63 ± 0.19	0.84 ± 0.32	0.195
Rotation (degrees)	0.005 ± 0.0015	0.006 ± 0.003	0.266

White matter tractography was performed using the software MRtrix (http://www.mrtrix.org). The fiber orientation density function (FOD) was estimated using constrained spherical deconvolution (Tournier et al., 2004) and tractography was performed using a deterministic algorithm. This choice was motivated by the fact that previous studies conducted by our group used deterministic tractography to perform whole brain tractography (Ottet et al., 2013b, Ottet et al., 2013a) and we wanted our studies to be comparable. An example of tractogram for one subject is displayed in supplementary Fig. 3.

The connectivity matrix was then built as an 83 × 83 matrix containing the number of streamlines connecting each pair of brain regions. The matrices were not thresholded, but were normalized by the average length of the streamlines connecting each pair of nodes (Hagmann et al., 2008).

#### 2.3.2 Computation of graph theory measures

Graph theory measures were computed starting from the connectivity matrices.

Network's measurements can be obtained on the weighted (if it contains the information about the number of streamlines) or binary (where only the existence of a connection between the pair of nodes is represented) connectivity matrices. Binary graphs give information about the core network organization, while weighted graphs also contain the information about the strength of the connection between two nodes. Either measures were included in this study.

*Global* and *local* networks characteristics were then computed using functions included in the Brain Connectivity Toolbox (brain-connectivity-toolbox.net). Global measures are computed over the entire connectivity matrix, while local measures are computed for each node of the network. The global measures included in this study were: *characteristic path length*, *efficiency*, *mean clustering coefficient*, *modularity* and *small world index.* The local measures included were: *betweenness centrality, clustering coefficient* and *strength.* A detailed description of each of these measures is provided in (Latora and Marchiori, 2001, Sporns et al., 2005, Sporns, 2006, Watts and Strogatz, 1998).

Briefly, the characteristic path length is the minimum number of edges connecting each pair of nodes in the network. The efficiency is the inverse of the mean path length, however, it is considered to be a more meaningful measure when computed on disconnected networks (Rubinov and Sporns, 2010). Modularity reflects the presence of sub-groups of nodes in the graph that are more densely connected to each other than to the rest of the network. The clustering coefficient of a node is the number of connections between the neighbors of that node. The average clustering coefficient therefore reflects how connectivity is clustered around each node. The small world index estimates the degree to which a network is a “small-world network”, meaning that is highly clustered while remaining highly efficient. The betweenness centrality is the number of short paths passing through a node, therefore, it reflects the path length (and the efficiency) at the nodal level and measures how central is each node in the network. Finally, the strength is the sum of the edges connecting a node.

#### 2.3.3 Computation of diffusion measures

Diffusion measures reflecting the microstructural integrity of white matter were computed with the TRACULA toolbox. This automatic algorithm performs probabilistic tractography, using a “ball-and-stick” model of diffusion (Behrens et al., 2007), to estimate the probability of 18 white matter pathways (Yendiki et al., 2011). TRACULA has the advantage of using cortical surfaces reconstructed with *freesurfer* for intra-subject registration. Furthermore, tractography is performed in the subjects' native space. One subject was excluded from the *psy+* group because of bad reconstruction of the tracts. Mean FA, AD and RD were computed for each fiber bundles.

#### 2.3.4 Statistical analyses

Univariate and multivariate analyses were conducted to investigate differences in the above-mentioned measures between patients with and without prodromal psychotic symptoms. The univariate analysis was performed with a Mann-Whitney *U* test in Matlab version 2014b, and False Discovery Rate (FDR) at a significance threshold of 0.05 was used to correct for multiple comparison. The multivariate analysis was conducted using functions included in the Pattern Recognition for Neuroimaging (PRONTO) toolbox (http://www.mlnl.cs.ucl.ac.uk/pronto/), with procedures similar to our previous work (Scariati et al., 2014). More in details, we used a naïve Bayes classifier using the “fitcnb” function of Matlab. The “Normal” option has been used to model for each feature a Gaussian distribution, while the other options have been left as default. The naïve Bayes classifier has been chosen to model more flexible decision boundaries (piecewise quadratic) than other linear classifiers such as Support Vector Machine (SVM). However, it should be noticed that the naïve Bayes approach, contrarily to SVM, assumes independency between the features, therefore, correlated features cannot be modeled. Nevertheless, the classifier will still benefit from correlated features because the decision will consider together the probabilities of different features. Therefore, if the probabilities go in the same direction, the decision will be reinforced. In addition, as mentioned in the introduction, we did not aim for generalizability in this study, but our purpose was to provide preliminary results and direct further investigations.

Accuracy was estimated using a leave-one-subject-out cross validation loop (LOOCV). At each loop, the exact prior probabilities for each class were 50.82% and 49.18%. The classifier was applied subsequently on 1) connectivity matrices containing the number of streamlines, 2) graph theory measures computed on the weighted graph, 3) graph theory measures computed on the binary graph and 4) diffusion measures. Global graph theory measures were entered individually in separated classifiers (5 classifiers). For each local measure, one classifier was built containing the measure for each region (*N* = 83 regions). For the diffusion measures, one classifier was built for FA, AD and RD separately, containing the mean value for this measure for each fiber bundle (*N* = 18 bundles).

The classification was then repeated after feature selection. Feature selection was performed with point biserial correlation to rank the features that showed the most important differences between the two groups. The classification was performed several times increasing the number of features kept. For the number of streamlines, accuracy was tested starting from the first 10 connections and adding 10 connections at the time until 1600. The global and local graph theory measures were put into one single matrix and accuracy was tested starting from the first 5 measures and adding 5 measures at the time until reaching the max number of features (*N* = 254). The measures iteratively added could be a global measure or a local measure of one specific brain region, based on the rank attributed during feature selection. For each of the diffusion measures, accuracy was tested by adding the mean value of each bundle one after the other until reaching 18. In order to evaluate the significance of the classification results, we used the Wilson's score interval to compute the accuracy 95% Confidence Interval (CI). Indeed, a significance level of 0.05 is achieved if the 95% confidence interval do not contain the chance value (Armitage et al., 2002, Moore, 2012), which is 50% for classification. The same approach has been used in our previously published study (Scariati et al., 2014).

To check for any residual effect of age, gender and type of scanner in the results, the analysis was repeated adding these variables as covariates. The correction was performed within the LOOCV loop, re-estimating at each loop the residuals in the training group and applying the regression to the test subject.

## 3 Results

### 3.1 Univariate analyses

There were no significant differences between *psy+* and *psy-* patients on any of the measures (i.e, the number of streamlines connecting pairs of brain regions, the graph theory measures and the diffusion measures).

### 3.2 Multivariate analysis

#### 3.2.1 Number of streamlines

**Fig. 2:**
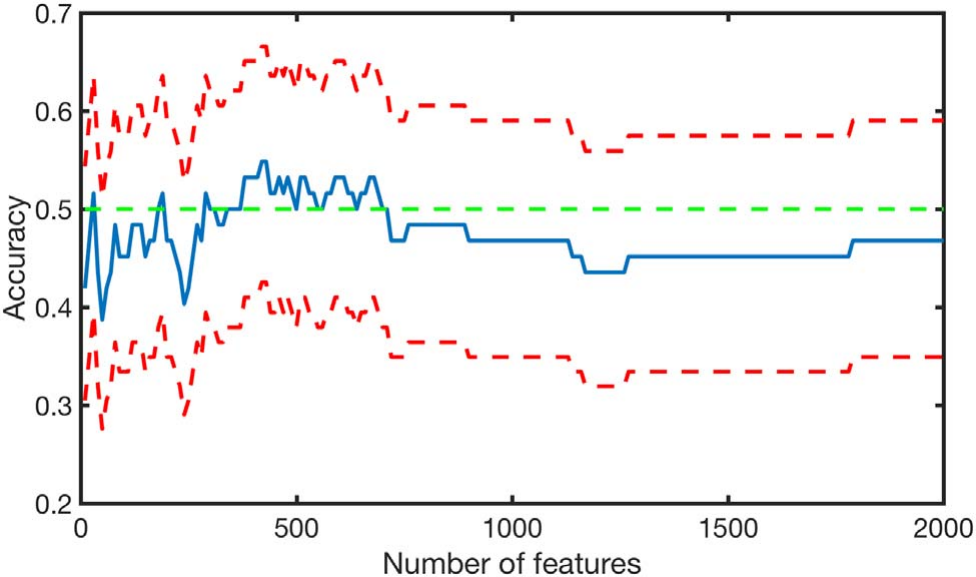
Accuracy plot for the number of streamlines. The blue line indicates the accuracy, the two red lines indicate the upper and lower confidence intervals. No significant accuracy was achieved at any features intervals. (For interpretation of the references to color in this figure legend, the reader is referred to the web version of this article.)

The connectivity matrix containing the number of streamlines connecting pairs of brain region did not provide a significant discrimination of patients with 22q11DS with and without psychotic symptoms. The accuracy achieved without features selection was 46.8% (CI: 34.9–59.0%). When feature selection was performed, no significant accuracy was achieved with any of the features (Fig. 2).

#### 3.2.2 Graph theory measures

**Fig. 3:**
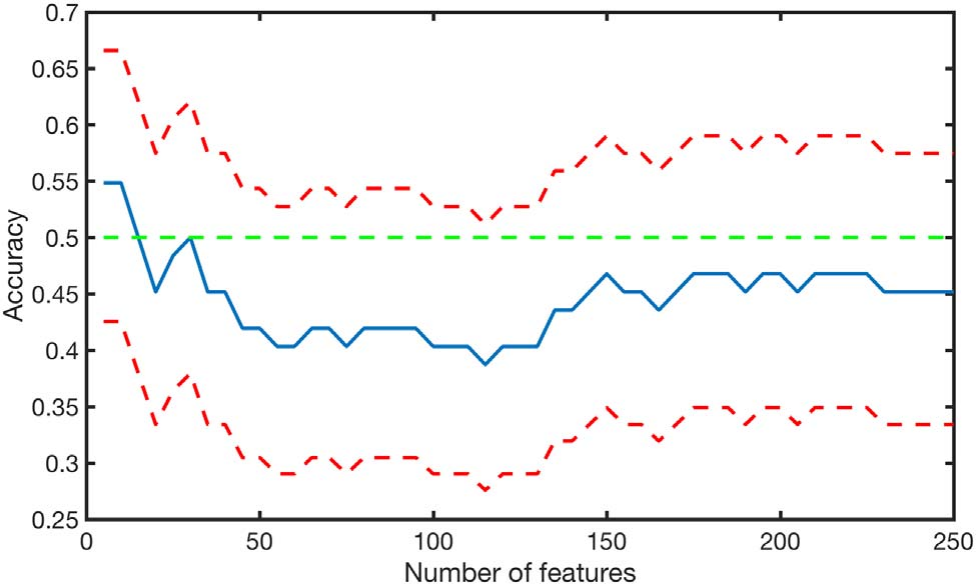
Accuracy plot of the discrimination analysis using graph theory measures computed on the weighted graph. Classification results when using all the graph theory measures and feature selection. The blue line indicates the accuracy, the two red lines indicate the upper and lower confidence intervals. No significant accuracy was achieved at any features intervals. (For interpretation of the references to color in this figure legend, the reader is referred to the web version of this article.)

No significant discriminations were obtained when using the weighted graph (Table 3, Fig. 3).

**Table 3:** Results of the classification analysis with global and local graph theory measures computed on the weighted graph.

	Accuracy with weighted graph
Characteristic path length	Accuracy = 54.8%, CI: 42.5–66.6%
	Sensitivity: 32.3%; Specificity: 77.4%
Efficiency	Accuracy = 58.1%, CI: 45.7–69.5%
	Sensitivity: 38.7%; Specificity: 77.4%
Mean clustering coefficient	Accuracy = 0.0%, CI: 0.0–5.8%
	Sensitivity: 0.0%; Specificity: 0.0%
Modularity	Accuracy = 48.4%, CI: 36.4–60.6%
	Sensitivity: 29.0%; Specificity: 67.7%
Small world index	Accuracy = 51.6%, CI: 39.4–63.6%
	Sensitivity: 74.2%; Specificity: 29.0%
Betweenness centrality	Accuracy = 35.5%, CI: 24.7–47.9%
	Sensitivity: 19.4%; Specificity: 51.6%
Clustering coefficient	Accuracy = 50.0%, CI: 37.9–62.1%
	Sensitivity: 32.3%; Specificity: 67.7%
Strength	Accuracy = 51.6%, CI: 39.4–63.6%
	Sensitivity: 29.0%; Specificity: 74.2%

**Fig. 4:**
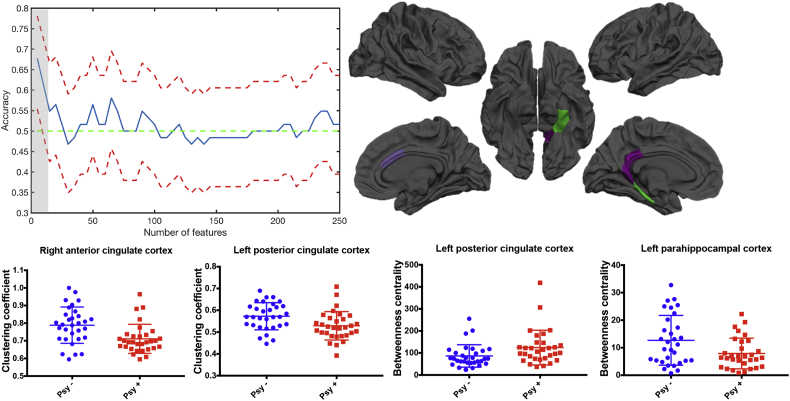
Results of the discrimination analysis using graph theory measures computed on the binary graph. On the top, the accuracy plot displays the maximum significant accuracy achieved with the five best features. The blue line indicates the accuracy and the red lines indicate the upper and lower confidence intervals. The brain maps show the regions where the betweenness centrality and the clustering coefficient contributed to the discrimination. The amygdala is not showed in the cortical maps as it is a sub-cortical region. The boxplots show the betweenness centrality and the clustering coefficient values for the corresponding regions. (For interpretation of the references to color in this figure legend, the reader is referred to the web version of this article.)

When using the binary graph, global and local measures did not discriminate patients with 22q11DS with prodromal psychotic symptoms (Table 4). However, when performing feature selection, the betweenness centrality and the clustering coefficient discriminated patients with and without psychotic symptoms (Fig. 4). Specifically, the maximum accuracy (67.7%, CI: 55.4–78.0%, sensitivity 67.7%, specificity 67.7%) was achieved with five features, which corresponded to the betweenness centrality in the right amygdala, left posterior cingulate (PCC) and the left parahippocampal cortices and the clustering coefficient in the right ACC and the left PCC (Fig. 4). To assess the stability of our results, we additionally performed leave one out bootstrapping, conducted by randomly removing one subjects from the *psy+* group and the correspondent matched subject in the *psy-* group. The results of this analysis have been reported in Supplementary Fig. 4. The accuracy remained higher than 50% in 100% of the trials and remained significant in 85% of the trials.

**Table 4:** Results of the classification analysis with global and local graph theory measures computed on the binary graph.

	Accuracy with binary graph
Characteristic path length	Accuracy = 48.4%, CI: 36.4–60.6%
	Sensitivity: 67.7%; Specificity: 29.0%
Efficiency	Accuracy = 46.8%, CI: 34.9–59.0%
	Sensitivity: 67.7%; Specificity: 25.8%
Mean clustering coefficient	Accuracy = 45.2%, CI: 33.4–57.5%
	Sensitivity: 48.4%; Specificity: 41.9%
Modularity	Accuracy = 50.0%, CI: 37.9–62.1%
	Sensitivity: 67.7%; Specificity: 32.3%
Small world index	Accuracy = 54.8%, CI: 42.5–66.6%
	Sensitivity: 74.2%; Specificity: 35.5%
Betweenness centrality	Accuracy = 50.0%, CI: 37.9–62.1%
	Sensitivity: 41.9%; Specificity: 58.1%
Clustering coefficient	Accuracy = 53.2%, CI: 41.0–65.1%
	Sensitivity: 48.4%; Specificity: 58.1%
Strength	Accuracy = 48.4%, CI: 36.4–60.6%
	Sensitivity: 45.2%; Specificity: 51.6%

These results remained significant also after covarying for age, gender and type of scanner. To take into account the effect of medication, we repeated the analysis after excluding the subjects under antipsychotics (*n* = 6). As reported in Supplementary Fig. 5 a significant discrimination was still obtained.

These findings suggest that rather than the absolute number of streamlines, differences in network architecture distinguish patients with and without psychotic symptoms.

#### 3.2.3 Diffusion measures

**Fig. 5:**
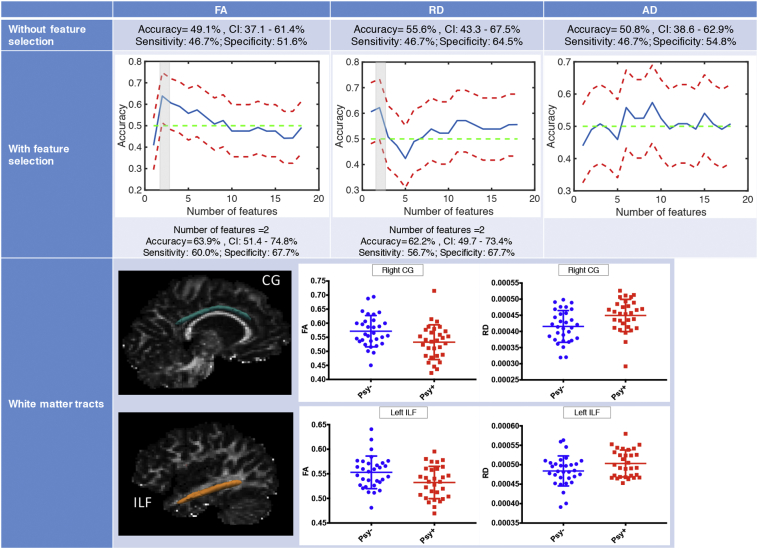
Results of the discrimination analysis using diffusion measures. In the first row, accuracy is reported for the multivariate analysis without features selection. In the second row, accuracy plots display the results when performing feature selection. The brain maps in the last row show the tracts that significantly discriminated patients with and without mild to attenuated psychotic symptoms. ILF = inferior longitudinal fasciculus, CG = cingulate gyrus, FA = Fractional Anisotropy, AD = axial diffusivity, RD = radial diffusivity.

When performing feature selection, the FA in the left inferior longitudinal fasciculus (ILF) and the right cingulate gyrus discriminated *psy+* and *psy-* patients (Fig. 5). Borderline accuracy was obtained also with the RD in the same tracts (Fig. 5). However, these results did not remain significant when correcting for age gender and scanner type.

## 4 Discussion

In this study, we investigated the association between white matter alterations and attenuated positive psychotic symptoms in a homogeneous group of patients carrying the same genetic deletion and matched for age, gender and IQ. The univariate comparison of connectivity measures between *psy+* and *psy-* patients did not give significant results. However, the multivariate analysis allowed us to discriminate patients with 22q11DS with and without attenuated positive psychotic symptoms using measures of network's structural architecture and white matter integrity.

In particular, when considering the binary graph, local network's properties, namely betweenness centrality and clustering coefficient, discriminated *psy+* from *psy-* patients with 22q11DS. Contrarily, we did not observe significant discriminations when considering the weighted graph, thus suggesting that local network alterations in patients with higher positive symptoms are related to the core organization of the connections, independently from their strength. Betweenness centrality reflects how much a node is relevant in the information flow within the network. Here, we found that betweenness centrality in the right amygdala, left PCC and the left parahippocampal cortex contributed to the classification of patients with 22q11DS with psychotic symptoms. In our previous study using similar methodologies to compare structural network integrity in patients with 22q11DS and healthy controls (Ottet et al., 2013b) we found a similar pattern of alterations in these regions. The results reported here complement these previous findings and suggest that the differences observed in patients with 22q11DS are driven by the most symptomatic patients and that reduced centrality of these nodes compromise the correct brain network's organization and contributes to the manifestation of psychotic symptoms.

Together with the reduced betweenness centrality, reduced clustering coefficient in the cingulate cortex contributed to the classification of psychotic patients with 22q11DS. In particular, disconnectivity of the ACC is one of the most replicated finding in patients with 22q11DS, and has been associated with psychotic symptoms severity (Rihs et al., 2013, Scariati et al., 2014, Sandini et al., 2017, Tomescu et al., 2014). Our results confirm that altered ACC connectivity may be a candidate predictor of psychosis development.

It is worth to notice, however, that no focal differences in these specific measures and regions were observed when using the univariate analysis, suggesting that there are not alterations in specific brain areas distinguishing patients with 22q11DS with higher psychotic symptoms. This means that the impairments we observed in the cingulate and parahippocampal cortices and in the amygdala are not independent from each other, but they contribute together to the discrimination of patients with 22q11DS with higher symptoms. The same consideration can be done for the measures that we found impaired, namely the betweenness centrality and the clustering coefficient, suggesting that the structural brain network is impaired at different levels in patients with higher symptoms scores. Interestingly, the pattern of regions we found to discriminate psy+ and psy- patients are part of the limbic system, thus indicating that altered connectivity within this system compromise the brain network's organization and is responsible for the manifestation of psychotic symptoms. The presence of impairments in limbic connections in patients with higher symptoms severity also emerged in our recent review of connectivity studies in 22q11DS (Scariati et al., 2016). Furthermore, alterations in the limbic system have extensively been reported in patients with schizophrenia (for instance (Bracht et al., 2014, Fitzsimmons et al., 2014, Oestreich et al., 2016) and, even if to a lesser extent, in UHR individuals and adolescents with schizotypal personality disorder (Rosell et al., 2014, Vijayakumar et al., 2016). Therefore, our findings suggest that alterations in limbic system are present in patients with 22q11DS with attenuated positive psychotic symptoms as in patients with schizophrenia and at risk of psychosis, thus representing a valuable biomarker for the development of a full-blown disorder.

In addition to alterations in the structural network, altered white matter microstructure predicted the presence of mild to severe psychotic symptoms. In particular, patients with 22q11DS expressing higher symptoms severity showed reduced FA and increased RD in the ILF and cingulate gyrus, thus indicating altered integrity of these tracts, which may reflect altered myelination. However, these results should be interpreted with caution as they did not remain significant after regression of age, gender and scanner type.

To date, only two studies used multivariate approaches to discriminate the presence of psychotic symptoms in patients with 22q11DS (Gothelf et al., 2011, Scariati et al., 2014). Gothelf et al. (2011) found that atypical patterns of white and grey matter maturation predicted increased severity of psychotic symptoms with high accuracy (~ 95%). In our previous study (Scariati et al., 2014), we were able to discriminate patients with and without prodromal psychotic symptoms using resting-state fMRI connectivity. In particular, disconnectivity in frontal brain regions and the ACC mainly contributed to the discrimination. Our results complement these previous findings and suggest that altered white matter architecture can also discriminate the presence of mild/severe psychotic symptoms in patients with 22q11DS.

However, in our study, a relatively low accuracy was achieved (67.7%) in the discrimination of patients with higher psychotic symptoms. This is similar to what have been observed in UHR populations, in which a lower discrimination accuracy was achieved (~ 65%, Pettersson-Yeo et al., 2013) compared to schizophrenia (Ardekani et al., 2011, Caan et al., 2006, Caprihan et al., 2008, Pettersson-Yeo et al., 2013). One reason that could explain this difference is that patients with schizophrenia are more symptomatic and have been affected by the disease for a long period of time. Therefore, this may have produced significant changes in their brains. On the opposite, brain alterations in patients with 22q11DS or in UHR individuals may be more subtle than the alterations observed in the full blown disease, but may be however important for predicting the patients outcome.

## 5 Limitations

This study presents a number of limitations. Some of them are related to the population being studied, such as the low sample size. This is especially true in light of the high dimensionality of our dataset. Furthermore, more than one classifier has been trained for the multivariate comparisons, which may have caused the detection of false positive results. Therefore, our results will need to be replicated in a larger sample of patients.

Furthermore, our groups of patients with and without prodromal psychotic symptoms presented psychiatric comorbidities and differed in terms of the use of psychotropic medication. However, our results remained significant after excluding the subjects that were taking antipsychotics at the time of testing, thus allowing us to partially exclude the effect of medication from our findings.

Another limitation is related to our DTI sequence, which does not allow an optimal reconstruction of crossing/kissing fibers. However, in order to reduce the duration of our scanning protocol, we have chosen to use a shorter sequence that is more suitable for children with psychiatric conditions and/or intellectual disability. This problem has however been partially solved using CSD for the FOD reconstruction and state of art methodologies for the preprocessing of DTI data. Furthermore, tractography reconstructions can be influenced by a number of factors, such as noisy data, that prevent us from affirming that the difference observed in this study are uniquely related to disconnectivity.

## 6 Conclusion

To conclude, our study represents the first attempt to distinguish patients with 22q11DS with and without attenuated positive psychotic symptoms using white matter connectivity and multivariate methodologies. Our results suggest that changes in structural network's properties may be candidate biomarkers of psychosis, which needs to be further explored by longitudinal investigations. Furthermore, the majority of patients included in the *psy+* group presented low levels of symptoms rather than a full-blown psychosis, thus indicating that network's structural alterations may be identified at an early stage in the psychosis development, which could permit a more effective prevention.

## Appendix A Supplementary data

Supplementary figures Image 1

## References

[bb0005] Ardekani B.A., Tabesh A., Sevy S., Robinson D.G., Bilder R.M., Szeszko P.R. (2011). Diffusion tensor imaging reliably differentiates patients with schizophrenia from healthy volunteers. Hum. Brain Mapp..

[bb0010] Armitage P., Berry G., Matthews J.n.s. (2002). Analysing means and proportions. Statistical Methods in Medical Research.

[bb0015] Armitage P.A., Bastin M.E. (2000). Selecting an appropriate anisotropy index for displaying diffusion tensor imaging data with improved contrast and sensitivity. Magn. Reson. Med..

[bb0020] Avants B.B., Tustison N.J., Song G., Cook P.A., Klein A., Gee J.C. (2011). A reproducible evaluation of ANTs similarity metric performance in brain image registration. NeuroImage.

[bb0025] Bammer R., Acar B., Moseley M.E. (2003). In vivo MR tractography using diffusion imaging. Eur. J. Radiol..

[bb0030] Bassett A.S., Chow E.W. (1999). 22q11 deletion syndrome: a genetic subtype of schizophrenia. Biol. Psychiatry.

[bb0035] Behrens T.E.J., Berg H.J., Jbabdi S., Rushworth M.F.S., Woolrich M.W. (2007). Probabilistic diffusion tractography with multiple fibre orientations: what can we gain?. NeuroImage.

[bb0040] Bracht T., Horn H., Strik W., Federspiel A., Razavi N., Stegmayer K., Wiest R., Dierks T., Müller T.J., Walther S. (2014). White matter pathway organization of the reward system is related to positive and negative symptoms in schizophrenia. Schizophr. Res..

[bb0045] Budde M.D., Kim J.H., Liang H.-F., Russell J.H., Cross A.H., Song S.-K. (2008). Axonal injury detected by in vivo diffusion tensor imaging correlates with neurological disability in a mouse model of multiple sclerosis. NMR Biomed..

[bb0050] Budde M.D., Xie M., Cross A.H., Song S.-K. (2009). Axial diffusivity is the primary correlate of axonal injury in the experimental autoimmune encephalomyelitis spinal cord: a quantitative pixelwise analysis. J. Neurosci..

[bb0055] Caan M.W.A., Vermeer K.A., van Vliet L.J., Majoie C.B.L.M., Peters B.D., den Heeten G.J., Vos F.M. (2006). Shaving diffusion tensor images in discriminant analysis: a study into schizophrenia. Med. Image Anal..

[bb0060] Caprihan A., Pearlson G.D., Calhoun V.D. (2008). Application of principal component analysis to distinguish patients with schizophrenia from healthy controls based on fractional anisotropy measurements. NeuroImage.

[bb0065] da Silva Alves F., Schmitz N., Bloemen O., van der Meer J., Meijer J., Boot E., Nederveen A., de Haan L., Linszen D., van Amelsvoort T. (2011). White matter abnormalities in adults with 22q11 deletion syndrome with and without schizophrenia. Schizophr. Res..

[bb0070] Daducci A., Gerhard S., Griffa A., Lemkaddem A., Cammoun L., Gigandet X., Meuli R., Hagmann P., Thiran J.-P. (2012). The connectome mapper: an open-source processing pipeline to map connectomes with MRI. PLoS One.

[bb0075] Dale A.M., Fischl B., Sereno M.I. (1999). Cortical surface-based analysis. I. Segmentation and surface reconstruction. NeuroImage.

[bb0080] Davatzikos C. (2004). Why voxel-based morphometric analysis should be used with great caution when characterizing group differences. NeuroImage.

[bb0085] DeRosse P., Nitzburg G.C., Ikuta T., Peters B.D., Malhotra A.K., Szeszko P.R. (2015). Evidence from structural and diffusion tensor imaging for frontotemporal deficits in psychometric schizotypy. Schizophr. Bull..

[bb0090] Desikan R.S., Ségonne F., Fischl B., Quinn B.T., Dickerson B.C., Blacker D., Buckner R.L., Dale A.M., Maguire R.P., Hyman B.T., Albert M.S., Killiany R.J. (2006). An automated labeling system for subdividing the human cerebral cortex on MRI scans into gyral based regions of interest. NeuroImage.

[bb0095] First M., Gibbon M., Spitzer R., Williams J., Benjamin L. (1996). Structured Clinical Interview for the DSM-IV Axis I Disorders (SCID-I).

[bb0100] Fitzsimmons J., Schneiderman J.S., Whitford T.J., Swisher T., Niznikiewicz M.A., Pelavin P.E., Terry D.P., Mesholam-Gately R.I., Seidman L.J., Goldstein J.M., Kubicki M. (2014). Cingulum bundle diffusivity and delusions of reference in first episode and chronic schizophrenia. Psychiatry Res. Neuroimaging.

[bb0105] Gothelf D., Hoeft F., Ueno T., Sugiura L., Lee A.D., Thompson P., Reiss A.L. (2011). Developmental changes in multivariate neuroanatomical patterns that predict risk for psychosis in 22q11.2 deletion syndrome. J. Psychiatr. Res..

[bb0110] Hagmann P., Cammoun L., Gigandet X., Meuli R., Honey C.J., Wedeen V.J., Sporns O. (2008). Mapping the structural Core of human cerebral cortex. PLoS Biol..

[bb0115] Hagmann P., Jonasson L., Maeder P., Thiran J.-P., Wedeen V.J., Meuli R. (2006). Understanding diffusion MR imaging techniques: from scalar diffusion-weighted imaging to diffusion tensor imaging and beyond. Radiogr. Rev. Publ. Radiol. Soc. N. Am. Inc.

[bb0120] Hagmann P., Thiran J.-P., Jonasson L., Vandergheynst P., Clarke S., Maeder P., Meuli R. (2003). DTI mapping of human brain connectivity: statistical fibre tracking and virtual dissection. NeuroImage.

[bb0125] Jalbrzikowski M., Villalon-Reina J.E., Karlsgodt K.H., Senturk D., Chow C., Thompson P.M., Bearden C.E. (2014). Altered white matter microstructure is associated with social cognition and psychotic symptoms in 22q11.2 microdeletion syndrome. Front. Behav. Neurosci..

[bb0130] Kates W.R., Olszewski A.K., Gnirke M.H., Kikinis Z., Nelson J., Antshel K.M., Fremont W., Radoeva P.D., Middleton F.A., Shenton M.E., Coman I.L. (2015). White matter microstructural abnormalities of the cingulum bundle in youths with 22q11.2 deletion syndrome: associations with medication, neuropsychological function, and prodromal symptoms of psychosis. Schizophr. Res..

[bb0135] Kaufman J., Birmaher B., Brent D., Rao U., Flynn C., Moreci P., Williamson D., Ryan N. (1997). Schedule for affective disorders and schizophrenia for school-age children-present and lifetime version (K-SADS-PL): initial reliability and validity data. J. Am. Acad. Child Adolesc. Psychiatry.

[bb0140] Kikinis Z., Cho K.I.K., Coman I.L., Radoeva P.D., Bouix S., Tang Y., Eckbo R., Makris N., Kwon J.S., Kubicki M., Antshel K.M., Fremont W., Shenton M.E., Kates W.R. (2016). Abnormalities in brain white matter in adolescents with 22q11.2 deletion syndrome and psychotic symptoms. Brain Imaging Behav..

[bb0145] Klauser P., Baker S.T., Cropley V.L., Bousman C., Fornito A., Cocchi L., Fullerton J.M., Rasser P., Schall U., Henskens F., Michie P.T., Loughland C., Catts S.V., Mowry B., Weickert T.W., Shannon Weickert C., Carr V., Lenroot R., Pantelis C., Zalesky A. (2016). White matter disruptions in schizophrenia are spatially widespread and topologically converge on brain network hubs. Schizophr. Bull..

[bb0150] Latora V., Marchiori M. (2001). Efficient behavior of small-world networks. Phys. Rev. Lett..

[bb0155] Maeder J., Schneider M., Bostelmann M., Debbané M., Glaser B., Menghetti S., Schaer M., Eliez S. (2016). Developmental trajectories of executive functions in 22q11.2 deletion syndrome. J. Neurodev. Disord..

[bb0160] Miller T.J., McGlashan T.H., Rosen J.L., Somjee L., Markovich P.J., Stein K., Woods S.W. (2002). Prospective diagnosis of the initial prodrome for schizophrenia based on the structured interview for prodromal syndromes: preliminary evidence of interrater reliability and predictive validity. Am. J. Psychiatry.

[bb0165] Moore D.S. (2012). Statistics, Concepts and Controversies (Loose Leaf).

[bb0170] Murphy K.C., Jones L.A., Owen M.J. (1999). High rates of schizophrenia in adults with velo-cardio-facial syndrome. Arch. Gen. Psychiatry.

[bb0175] O'Connor S., Agius M. (2015). A systematic review of structural and functional MRI differences between psychotic and nonpsychotic depression. Psychiatr. Danub..

[bb0180] Oestreich L.K.L., Pasternak O., Shenton M.E., Kubicki M., Gong X., McCarthy-Jones S., Whitford T.J. (2016). Abnormal white matter microstructure and increased extracellular free-water in the cingulum bundle associated with delusions in chronic schizophrenia. NeuroImage.

[bb0185] Ottet M.-C., Schaer M., Cammoun L., Schneider M., Debbané M., Thiran J.-P., Eliez S. (2013). Reduced fronto-temporal and limbic connectivity in the 22q11.2 deletion syndrome: vulnerability markers for developing schizophrenia?. PLoS One.

[bb0190] Ottet M.-C., Schaer M., Debbané M., Cammoun L., Thiran J.-P., Eliez S. (2013). Graph theory reveals dysconnected hubs in 22q11DS and altered nodal efficiency in patients with hallucinations. Front. Hum. Neurosci..

[bb0195] Perlstein M.D., Chohan M.R., Coman I.L., Antshel K.M., Fremont W.P., Gnirke M.H., Kikinis Z., Middleton F.A., Radoeva P.D., Shenton M.E., Kates W.R. (2014). White matter abnormalities in 22q11.2 deletion syndrome: preliminary associations with the Nogo-66 receptor gene and symptoms of psychosis. Schizophr. Res..

[bb0200] Pettersson-Yeo W., Allen P., Benetti S., McGuire P., Mechelli A. (2011). Dysconnectivity in schizophrenia: where are we now?. Neurosci. Biobehav. Rev..

[bb0205] Pettersson-Yeo W., Benetti S., Marquand A.F., Dell'Acqua F., Williams S.C.R., Allen P., Prata D., McGuire P., Mechelli A. (2013). Using genetic, cognitive and multi-modal neuroimaging data to identify ultra-high-risk and first-episode psychosis at the individual level. Psychol. Med..

[bb0210] Radoeva P.D., Bansal R., Antshel K.M., Fremont W., Peterson B.S., Kates W.R. (2016). Longitudinal study of cerebral surface morphology in youth with 22q11.2 deletion syndrome, and association with positive symptoms of psychosis. J. Child Psychol. Psychiatry.

[bb0215] Reich W. (2000). Diagnostic interview for children and adolescents (DICA). J. Am. Acad. Child Adolesc. Psychiatry.

[bb0220] Rihs T.A., Tomescu M.I., Britz J., Rochas V., Custo A., Schneider M., Debbané M., Eliez S., Michel C.M. (2013). Altered auditory processing in frontal and left temporal cortex in 22q11.2 deletion syndrome: a group at high genetic risk for schizophrenia. Psychiatry Res. Neuroimaging.

[bb0225] Rosell D.R., Futterman S.E., McMaster A., Siever L.J. (2014). Schizotypal personality disorder: a current review. Curr. Psychiatry Rep..

[bb0230] Rubinov M., Sporns O. (2010). Complex network measures of brain connectivity: uses and interpretations. NeuroImage.

[bb4000] Sandini C., Scariati E., Padula M.C., Schneider M., Schaer M., Van De Ville D., Eliez S. (2017). Cortical dysconnectivity measured by structural covariance is associated with the presence of psychotic symptoms in 22q11.2 deletion syndrome. Biol. Psychiatry Cogn. Neurosci. Neuroimaging.

[bb0235] Scariati E., Padula M.C., Schaer M., Eliez S. (2016). Long-range dysconnectivity in frontal and midline structures is associated to psychosis in 22q11.2 deletion syndrome. J. Neural Transm..

[bb0240] Scariati E., Schaer M., Richiardi J., Schneider M., Debbané M., Van De Ville D., Eliez S. (2014). Identifying 22q11.2 deletion syndrome and psychosis using resting-state connectivity patterns. Brain Topogr..

[bb0245] Schaer M., Debbané M., Bach Cuadra M., Ottet M.-C., Glaser B., Thiran J.-P., Eliez S. (2009). Deviant trajectories of cortical maturation in 22q11.2 deletion syndrome (22q11DS): a cross-sectional and longitudinal study. Schizophr. Res..

[bb0250] Schneider M., Debbané M., Bassett A.S., Chow E.W., Fung W.L.A., van den Bree M.B., Owen M., Murphy K.C., Niarchou M., Kates W.R. (2014). Psychiatric disorders from childhood to adulthood in 22q11. 2 deletion syndrome: results from the International Consortium on Brain and Behavior in 22q11. 2 Deletion Syndrome. Am. J. Psychiatry.

[bb0255] Schneider M., Schaer M., Mutlu A.K., Menghetti S., Glaser B., Debbané M., Eliez S. (2014). Clinical and cognitive risk factors for psychotic symptoms in 22q11.2 deletion syndrome: a transversal and longitudinal approach. Eur. Child Adolesc. Psychiatry.

[bb0260] Song S.-K., Sun S.-W., Ju W.-K., Lin S.-J., Cross A.H., Neufeld A.H. (2003). Diffusion tensor imaging detects and differentiates axon and myelin degeneration in mouse optic nerve after retinal ischemia. NeuroImage.

[bb0265] Sporns O. (2006). Small-world Connectivity, Motif Composition, and Complexity of Fractal Neuronal Connections. Biosystems, Dedicated to the Memory of Ray Paton.

[bb0270] Sporns O., Tononi G., Kötter R. (2005). The human connectome: a structural description of the human brain. PLoS Comput. Biol..

[bb0275] Sundram F., Campbell L.E., Azuma R., Daly E., Bloemen O.J.N., Barker G.J., Chitnis X., Jones D.K., van Amelsvoort T., Murphy K.C., Murphy D.G.M. (2010). White matter microstructure in 22q11 deletion syndrome: a pilot diffusion tensor imaging and voxel-based morphometry study of children and adolescents. J. Neurodev. Disord..

[bb0280] Tomescu M.I., Rihs T.A., Becker R., Britz J., Custo A., Grouiller F., Schneider M., Debbané M., Eliez S., Michel C.M. (2014). Deviant dynamics of EEG resting state pattern in 22q11.2 deletion syndrome adolescents: a vulnerability marker of schizophrenia?. Schizophr. Res..

[bb0285] Tournier J.-D., Calamante F., Gadian D.G., Connelly A. (2004). Direct estimation of the fiber orientation density function from diffusion-weighted MRI data using spherical deconvolution. NeuroImage.

[bb0290] van den Heuvel M.P., Sporns O., Collin G., Scheewe T., Mandl R.C.W., Cahn W., Goñi J., Hulshoff Pol H.E., Kahn R.S. (2013). Abnormal Rich Club Organization and functional brain dynamics in schizophrenia. JAMA Psychiat..

[bb0295] Váša F., Griffa A., Scariati E., Schaer M., Urben S., Eliez S., Hagmann P. (2016). An affected core drives network integration deficits of the structural connectome in 22q11.2 deletion syndrome. NeuroImage.

[bb0300] Vijayakumar N., Bartholomeusz C., Whitford T., Hermens D.F., Nelson B., Rice S., Whittle S., Pantelis C., McGorry P., Schäfer M.R., Amminger G.P. (2016). White matter integrity in individuals at ultra-high risk for psychosis: a systematic review and discussion of the role of polyunsaturated fatty acids. BMC Psychiatry.

[bb0305] Watts D.J., Strogatz S.H. (1998). Collective dynamics of “small-world” networks. Nature.

[bb0310] Wechsler D. (2008). Wechsler Adult Intelligence Scale—4th Edition (WAIS-4).

[bb0315] Wechsler D. (2004). The Wechsler Intelligence Scale for Children—Fourth Edition.

[bb0320] Wechsler D. (1997). Wechsler Adult Intelligence Scale—3rd Edition (WAIS-3).

[bb0325] Wechsler D. (1991). The Wechsler Intelligence Scale for Children—Third Edition.

[bb0330] Yendiki A., Koldewyn K., Kakunoori S., Kanwisher N., Fischl B. (2014). Spurious group differences due to head motion in a diffusion MRI study. NeuroImage.

[bb0335] Yendiki A., Panneck P., Srinivasan P., Stevens A., Zöllei L., Augustinack J., Wang R., Salat D., Ehrlich S., Behrens T., Jbabdi S., Gollub R., Fischl B. (2011). Automated probabilistic reconstruction of white-matter pathways in health and disease using an atlas of the underlying anatomy. Front. Neuroinform..

[bb0340] Zalesky A., Fornito A., Seal M.L., Cocchi L., Westin C.-F., Bullmore E.T., Egan G.F., Pantelis C. (2011). Disrupted axonal fiber connectivity in schizophrenia. Biol. Psychiatry.

[bb0345] Zhong J., Wu S., Zhao Y., Chen H., Zhao N., Zheng K., Zhao Z., Chen W., Wang B., Wu K. (2013). Why psychosis is frequently associated with Parkinson's disease?. Neural Regen. Res..

