# Supplementary material for: Altered structural network architecture is predictive of the presence of psychotic symptoms in patients with 22q11.2 deletion syndrome
Source: Neuroimage Clin. 2017 Jul 26;16:142–50. doi: 10.1016/j.nicl.2017.07.023 (PMC5540832; doi:10.1016/j.nicl.2017.07.023)

**Supplementary figure 1.** Distribution of positive symptoms severity among *psy+* patients for each SIPS subscale score. P1=Unusual thoughts/delusional ideas, P2=Suspiciousness/persecutory ideas, P3=Grandiosity, P4=Perceptual abnormalities/hallucinations, P5=Disorganized communication.

**Supplementary figure 2.** Age and gender distribution in patients with (*psy+*) and without (*psy-*) attenuated positive psychotic symptoms.

**Supplementary figure 3.** Example of tractogram for one subject.


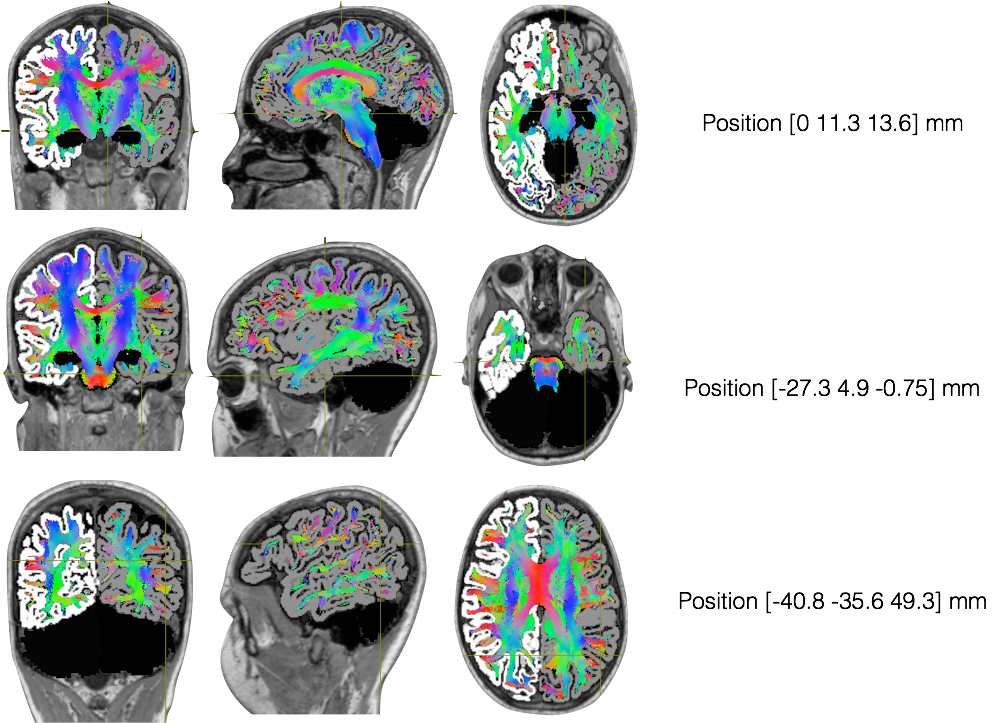


**Supplementary figure 4**. Results of the leave one out bootstrapping analysis performed by removing one subjects from the psy+ group and the correspondent matched subject in the psy- group for a total of 31 trials. The blue line indicates the accuracy, while the red lines indicate the lower and upper confidence interval.


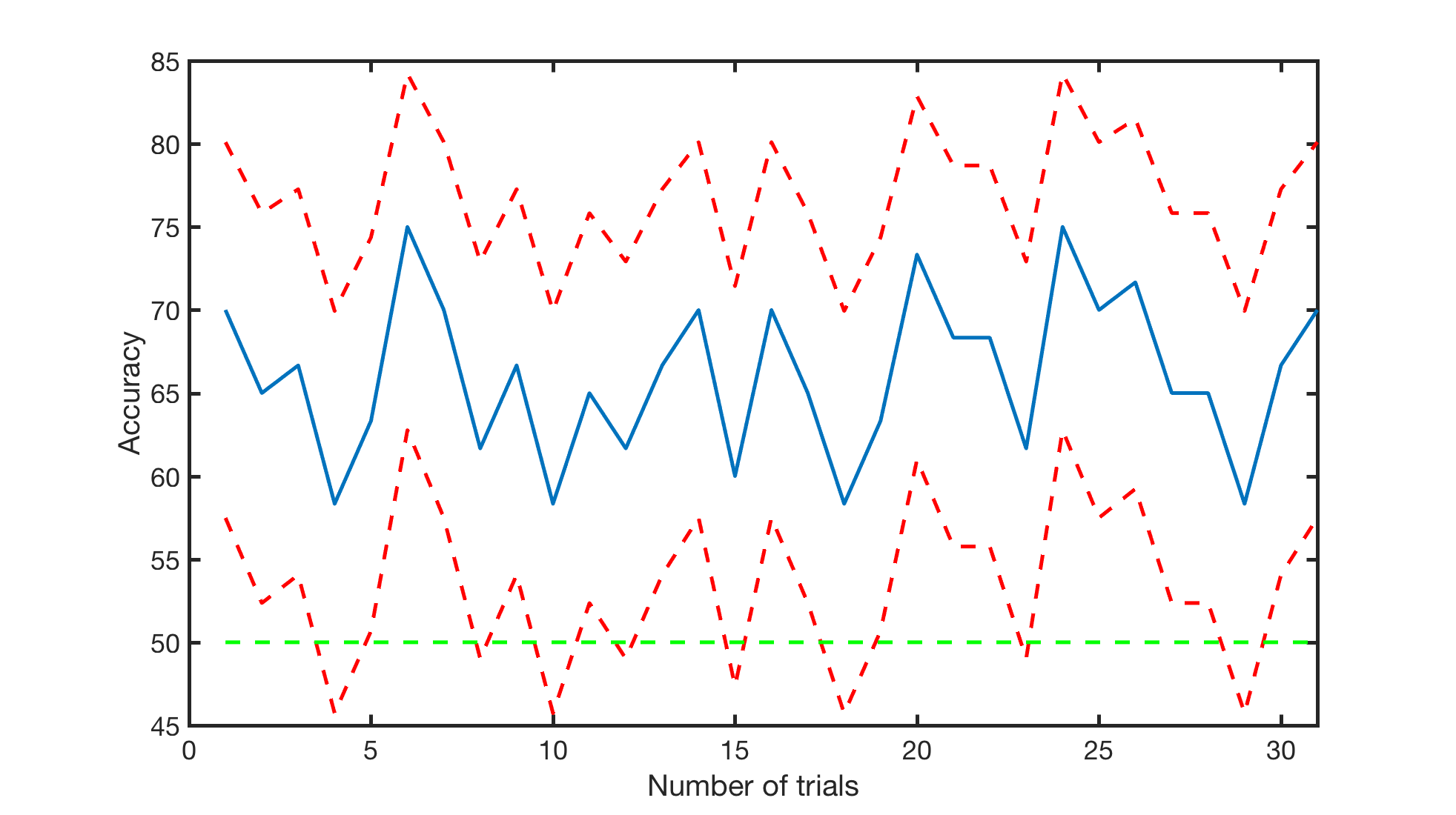


**Supplementary figure 5**. Results of the discrimination analysis using graph theory measures computed on the binary graph after removing the subjects taking psychotropic medication. A successful discrimination (Accuracy= 70.0%, CI: 56.2-80.9%, sensitivity: 56.0%, Specificity: 84.0%) was obtained with 25 features, which included the 5 features that significantly discriminated the original groups of subjects. The blue line indicates the accuracy, while the red lines indicate the lower and upper confidence interval.


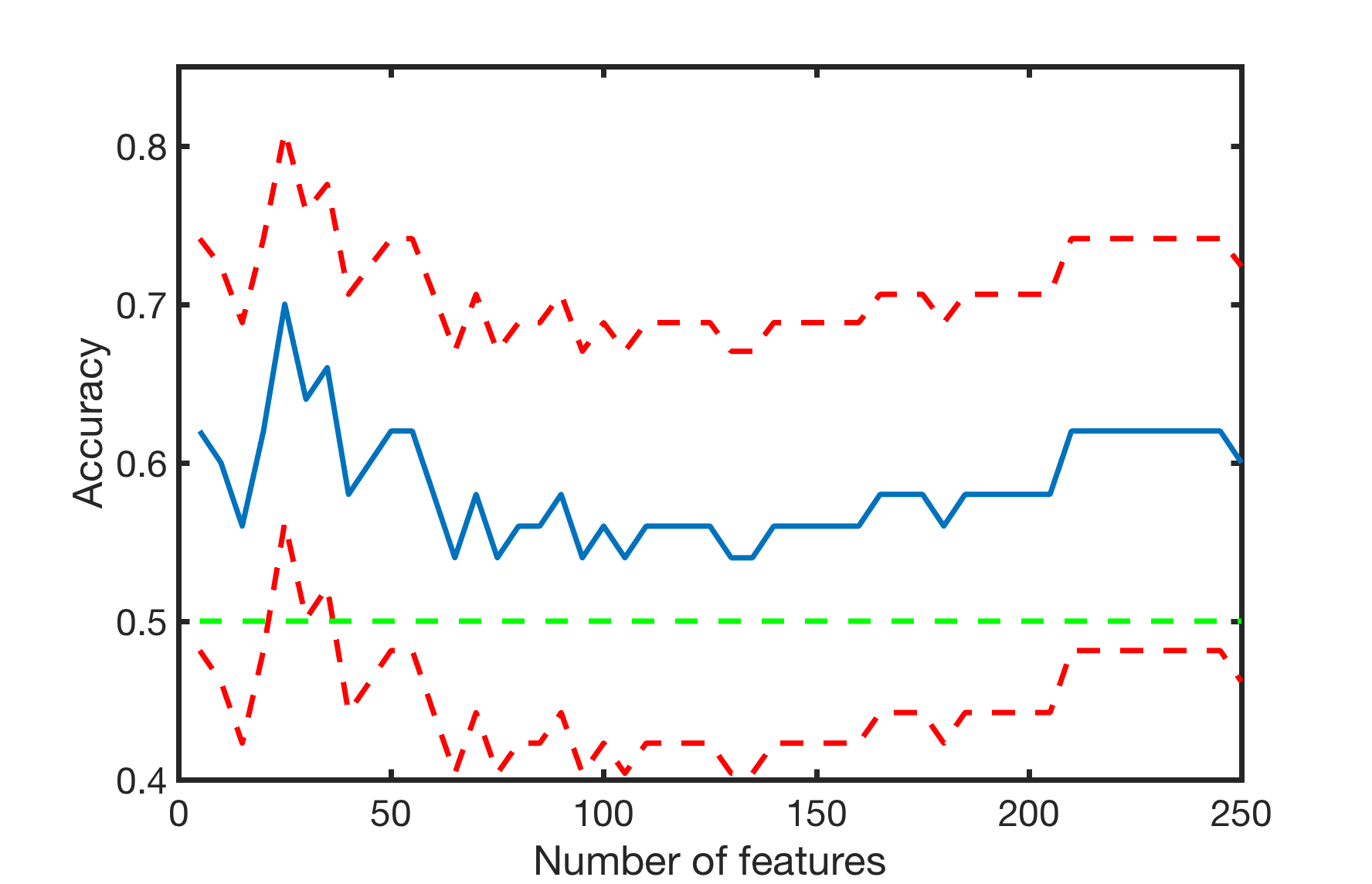

Supplement: Supplementary file 1 — Supplementary figures [file mmc1.docx]
